# The effect of dietary protein levels on body weight gain, carcass production, nitrogen emission, and efficiency of productions related to emissions in thin-tailed lambs

**DOI:** 10.14202/vetworld.2019.72-78

**Published:** 2019-01-15

**Authors:** Ari Prima, Endang Purbowati, Edy Rianto, Agung Purnomoadi

**Affiliations:** 1 Department of Animal Sciences, Faculty of Animal Agricultural Sciences, Diponegoro University, Tembalang campus, Semarang, 50275, Indonesia; 2 Department of Animal Science, Faculty of Agriculture and Animal Sciences, University of Muhammadiyah Malang, Jalan Raya Tlogomas 246, Malang, 65144, Indonesia

**Keywords:** average daily gain, carcass productions, crude protein levels, efficiency emissions to productions, nitrogen emission, nitrous oxide emission

## Abstract

**Aim:**

This study was aimed to evaluate dietary crude protein (CP) level on performance of body weight (BW) gain, carcass production, and nitrogen emission on lambs.

**Materials and Methods:**

A total of 12 male thin-tailed lambs (15.2±1.8 kg initial BW and aged 3-4 months) were assigned to completely randomized design for 84-day feeding trial. The animals were divided into three different levels of CP (i.e., 14%, 16%, and 18% with isocaloric diets and 60% total digestible nutrients) with four replications.

**Results:**

Increasing CP level was not significantly affected on average daily gain (ADG), carcass production, N and N_2_O emissions, and efficiency of emissions related to the productions. The average of ADG, carcass production, meat production, meat protein production, N emission, and N_2_O emission was 141.4 g, 11.6 kg, 6.8 kg, 0.9 kg, 53.1 g/day, and 0.3 g/day, respectively. The efficiency of ADG, carcass production, meat production, and meat protein related to N emissions were 119.7 g/kg, 4.4 g/kg, 2.5 g/kg, and 56.6 g/kg, respectively, while N_2_O emissions related to ADG, carcass production, meat production, and meat protein were 2.4 g/kg, 0.027 g/kg, 0.36 g/kg, and 0.34 g/kg, respectively.

**Conclusions:**

It can be concluded that the increase of CP level up to 18% did not affect productivity, N emissions, and efficiency of emissions per unit product because the increase of CP was not balanced by energy content in feed.

## Introduction

Fattening lambs were aimed to obtain high slaughter and carcass weight. Protein is one of the nutrients that affect lambs’ growth and slaughter weight (SW) [1]. Several studies reported that increasing crude protein (CP) levels resulted in increased DM intake [2-4], increased average daily gain (ADG) [2-5], increased carcass weight [4,6], and increased nitrogen excretions [3,5,7]. However, on the other hand, increasing CP levels did not effect on ADG [6-9], carcass weight [3,8,9], and nitrogen excretions [8].

Those studies have not considered nitrogen emissions [2,4,9], whereas other studies have observed the nitrogen emissions [3,5-8], but they have not calculated the efficiency of nitrogen emissions to productivity. The importance of calculating the efficiency of production related to the nitrogen emissions is because the increase of protein level from 13% to 17% did not only affect on ADG but also resulted in high nitrogen emissions [8]. This result showed that the ratio of nitrogen emissions per ADG might also linearly increase with protein level.

The effect of protein levels have to be clarified in ADG, carcass productions and related to nitrogen (N) emissions [8]. Therefore, this study was aimed to evaluate dietary protein levels on performance of body weight (BW) gain, carcass production, and nitrogen emission on lambs.

## Materials and Methods

The experiment was conducted at the Research Farm of Meat and Dairy Production Laboratory, Department of Animal Science, Diponegoro University, Semarang, Indonesia, from February to July 2016.

### Ethical approval

Using Animal and Scientific Procedures in this study has been approved by Animal Ethics Committee in Faculty of Animal and Agricultural Science, Diponegoro University, Indonesia.

### Animals, experimental design, and treatments

Twelve male thin-tailed lambs with average BW of 15.2±1.8 kg and aged 3-4 of months were assigned to completely randomized design for 84-day feeding trial. The animals were divided into three different levels of CP (i.e., 14%, 16%, and 18%) with four replications. Ingredients and chemical compositions of experimental diets are shown in Table-1.

**Table-1:** Dietary ingredients and nutrient content of experimental diets (DM basis).

Item	Treatment		

	14% CP	16% CP	18% CP
Ingredients			
Sugarcane top, %	30.2	29.0	28.5
Cassava peel, %	15.0	15.0	15.0
Rice bran, %	18.0	16.0	14.0
Cassava flour, %	11.5	9.5	7.0
Soybean meal, %	13.5	17.5	21.5
Fish meal, %	3.8	5.0	6.0
Sugarcane molasses, %	6.0	6.0	6.0
Mineral, %	2.0	2.0	2.0
Chemical compositions			
DM, %	79.4	81.7	81.1
OM, % DM	71.5	71.9	72.0
CP, % DM	13.3	15.5	17.6
EE, % DM	3.9	3.7	3.5
NDF, % DM	54.6	52.7	51.2
ADF, % DM	34.5	33.3	32.6
NFE, % DM	39.2	38.1	36.7
TDN, % DM	60.3	61.1	61.6
ME, MJ kg	10.5	10.6	10.7

DM=Dry matter, CP=Crude protein, OM=Organic matter, NDF=Neutral detergent fiber, ADF=Acid detergent fiber, NFE=Nitrogen free extract, TDN=Total digestible nutrients, ME=Metabolizable energy, MJ=Mega Joule

### Animal feeding and management

The lambs were raised in individual pen. Feed and water were given *ad libitum*. The diet was offered to the animals every morning at 06:00 am. The refusal is collected and weighed in the morning before new feed was given. Feed offered and refusal were measured daily, and feed intake was calculated as the difference between two measurements. The lambs were weighed every week in the morning before feeding to evaluate growth performance.

### Digestion trial, chemical analysis, and nitrous oxide measurement

Digestion trial was conducted for 7 days using total collection methods. The amounts of feed offered, feed refused, feces, and urine from each animal were weighed and recorded daily. During total collection, 100 g of samples of each feed offered and feed refused were sampled from individual lambs. Approximately 500 g of feces and 500 ml of urine were sampled and stored at 18°C every day during total collection. The final pH of the urine was maintained below 3. About 10% of the daily feces and urine were sampled for each animal in a deep freezer (−20°C) for chemical analysis.

The dry matter (DM), CP, ether extract (EE), and ash in feed and feces were analyzed following the Association of Official Analytical Chemists [10]. The nitrogen (N) content of feed, feces, and urine was determined by Kjeldahl method [10]. Neutral detergent fiber (NDF) and acid detergent fiber (ADF) were determined by Van Soest method [11]. Allantoin (purine derivatives) in urine was determined by Young and Conway method using Spectrophotometer (Shimadzu, Japan) at wavelength 522 nm. This value was then used to calculate purine derivative value for estimating microbial protein synthesis using Chen and Gomes equation for sheep [12]: Y = 0.84X + (0.150 LW^0.75^e^−0.25X^) where Y is allantoin (representing purine derivatives excretion; mmol/day), X corresponds to the absorbed microbial purines (mmol/day), and LW^0.75^. The intestinal flow of microbial N (g/day) was estimated based on the amount of microbial purines that were absorbed (X mmol/day), according to the equation of Chen and Gomes . [12]: MN (g/day) = X (mmol/day) ×70/0.116×0.83×1000; or MN (g/day) = 0.727X (mmol/day), assuming a digestibility of 0.83 for microbial purines, a ratio of 0.116 for purine N: total N, and a N content of purines of 70 mgN/mmol.

Nitrous oxide (N_2_O g/day) excreted in feces and urine was calculated according to the guidelines of IPCC [13], where 2% of the N excreted in livestock manure (feces and urine) was the emission factor that was adopted to find the amount of N_2_O emitted. The amount of N in each feces and urine (g/day) was calculated by multiplying N content (%) in each feces and urine with the weight (g/day) of feces and urine, respectively. Then, the N of feces and urine was summed to obtain a total of N excreted (g/day).

### Slaughtering and carcassing

The lambs were slaughtered after 84 days of feeding trial. Lambs were fasted 6 h before slaughtered. The slaughter method was followed by *halal* method. Lambs were skinned, and the visceral organs were separated and weighed. Lambs were skinned, and the visceral organs were separated for obtained carcass and each part was weighed. The carcass (kidneys and internal fat included) were chilled in a cold room at 18°C for 8 h, then weighed. Each carcass was split along the vertebrae into two halves. Carcass was separated into meat, fat, and bone, then weighed, and reported as a percentage of the cold carcass weight (CCW). The number (kg) meat protein was measured from the average of the percentage meat protein in *Bicep femoris* and *Longissimus dorsi* muscle multiplied with carcass meat (kg).

### Nitrogen and nitrous oxide emissions related to productivity

The efficiency of production to the emissions was measured by dividing the amount of N excretion (g/day) to ADG (g/day), carcass (kg) production, meat (kg) production, and kg meat protein and so for N_2_O emissions (g/day).

### Statistical analysis

All data observed were analyzed using analysis of variance according to Gomez and Gomez [14]. The level of significance was determined at 5%. If there was significance, then analyzed by Duncan multiple range test.

## Results

### ADG and carcass production

The data of ADG and carcass production are shown in Table-2. The effect of CP level was not significant (p>0.05) on ADG, feed conversion, SW, hot carcass weight (HCW), CCW, non-carcass weight, hot carcass yield (HCY) and cold carcass yield (CCY), meat, fat, and bone weight, meat-fat ratio, meat-bone ratio, and meat-protein content.

**Table-2:** ADG and carcass production.

Item	Treatment			SEM	p-value

	CP 14%	CP 16%	CP 18%		
Lambs performance					
ADG, g	138.9	151.1	134.4	13.04	0.447
FC, g DMI/g ADG	9.2	8.6	9.5	1.07	0.705
Carcass production					
Slaughter weight, kg	24.8	26.5	26.0	2.10	0.737
HCW, kg	11.7	12.6	12.7	1.38	0.735
CCW, kg	11.0	11.9	12.0	1.26	0.688
Non carcass, kg	13.1	13.9	13.3	0.81	0.664
Meat, kg	6.4	7.0	7.0	0.77	0.735
Total fat, kg	2.2	2.6	2.6	0.41	0.527
Bone, kg	2.3	2.3	2.4	0.21	0.910
HCY, %	46.7	47.5	48.8	1.84	0.564
CCY, %	43.8	44.9	45.9	1.62	0.463
Non carcass, %	53.3	52.5	51.2	1.84	0.564
Meat, % SW	25.8	26.4	26.9	1.55	0.976
Total fat, % SW	8.8	9.8	10.0	2.07	0.536
Bone, % SW	9.2	8.6	9.2	1.63	0.411
Meat/fat, kg/kg	2.9	2.6	2.8	0.32	0.626
Meat/bone, kg/kg	2.7	3.0	2.9	0.24	0.502
Meat protein content, %					
*Bicep femoris*	13.5	14.3	15.0	1.20	0.472
*Longissimus dorsi*	15.1	15.4	13.1	1.11	0.131
Meat protein, kg	0.9	1.0	1.0	0.14	0.717

SEM=Standard error of means, ADG=Average daily gain, FC=Feed cost, HCW=Hot carcass weight, CCW=Cold carcass weight, HCY=Hot carcass yield, CCY=Cold carcass yield, SW=Slaughter weight

### Nutrients’ intake and digestibility

The intake and digestibility of DM, OM, CP, EE, NDF, ADF, and NFE are shown in Table-2. The intake and digestibility of DM, OM, EE, NDF, ADF, and NFE were similar among treatments, except for CP in which 18% of CP had the highest CP intake and digestibility (p<0.05).

### Nitrogen balance and microbial efficiency

The data of N balance and microbial efficiency are presented in Table-3. N intake and N retention of lambs fed 18% CP had the highest values among treatments (p<0.05), while the N excretion in feces and urine was similar (p>0.05). The highest percentage of N feces was found in 14% of CP level (p<0.05). There was no effect (p>0.05) of CP levels on urine excretion, allantoin excretion, purine derivative excretion, microbial nitrogen synthesis, and its efficiency (Table-4).

**Table-3:** Nitrogen balance.

Item	Treatment			SEM	p-value

	CP 14%	CP 16%	CP 18%		
N intake, g/day	27.2^b^	32.2^ab^	35.8^a^	2.80	0.040
Fecal N, g/day	8.6	9.6	8.3	0.93	0.396
Urine N, g/day	6.5	8.6	8.1	1.46	0.373
Total N emission, g/day	15.1	18.2	16.4	1.96	0.322
N retention, g/day	12.1	13.8	19.3	2.62	0.052
Fecal N, %	31.9^a^	30.4^a^	23.2^b^	3.14	0.048
Urine N, %	24.1	27.4	22.4	4.63	0.572
Total N emission, %	56.0	57.8	45.7	6.33	0.174
N retention, %	44.0	42.2	54.3	6.33	0.174

SEM=Standard error of means, N=Nitrogen.

a,b Means with different superscripts in the same row differ significantly (p<0.05)

**Table-4:** Daily urine excretion, allantoin excretion, purine derivative excretion, absorbed purine, microbial crude protein, and microbial efficiency.

Item	Treatment			SEM	p-value

	CP 14%	CP 16%	CP 18%		
Urine excretion, L	1.19	1.16	1.13	0.027	0.868
Allantoin, mmol/day	6.56	7.77	7.97	10.78	0.809
PDE, mmol/day	7.71	9.14	9.38	14.92	0.809
AP, mmol/day	8.73	10.53	10.87	3.48	0.809
MCP, g/day	39.67	47.84	49.39	15.85	0.809
ME, gMN/kgOM	14.67	17.52	9.89	6.44	0.514

SEM=Standard error of means, PDE=Purine derivative excretion, AP=Absorbed purine, MCP=Microbial crude protein, ME=Microbial efficiency

### ADG, carcass production, and meat productions related to nitrous oxide emissions

The data of N and N_2_O emissions are presented in Table-5. The effect of CP levels was not significant on N_2_O emissions in feces and urine (p>0.05). The data of ADG, carcass production, and meat production related to N and N_2_O emissions are presented in Table-6. The ADG, carcass production, and meat production related to the emissions of N and N_2_O were not significant among treatments (p>0.05).

**Table-5:** Nitrous oxide emissions.

Item	Treatment			SEM	p-value

	CP 14%	CP 16%	CP 18%		
Fecal N_2_O, g/day	0.17	0.19	0.17	0.01	0.405
Urinary N_2_O, g/day	0.13	0.17	0.16	0.02	0.379
Total N_2_O, g/day	0.30	0.33	0.36	0.04	0.368

SEM=Standard error of means, DMI=Dry matter intake, CH_4_=Methane, N_2_O=Nitrous oxide

## Discussion

### ADG and carcass production

Lambs need protein to grow [1], but in this study, the increase of CP level did not increase ADG, SW, HCW, CCW, HCY, and CCY. The differences in CP intake among treatments were over 20 g, and according to nutrients’ requirement standard of Kearl [15], lambs should be able to give a 75 g/day the difference of ADG. However, in this study, the lambs were only able to give 10-15 g/day of ADG, and statistically, the ADG was not different. It is indicated that the CP level was not linear with ADG. In this study, the increase of CP level increased N intake but was not affected to N emissions (g/day) in feces and urine. Theoretically, this condition will differ the N retention. The data on Table-3 showed that the increase of CP level numerically increased N retention (12.1 vs. 13.8 vs. 19.3 g/day for 14, 16, and 18% CP, respectively) but failed to reach a significant level (p=0.052). Non-significant of N retention was the reason for the non-significant of the ADG.

As mentioned above, the increase of CP level was not able to increase the ADG, and therefore, the SW, CW, and meat protein content were similar. The similarity SW will produce a similar CW since the SW affects the carcass weight of 88-90% [16]. In addition, CP levels only have a minor effect on carcass weight and percentage in lambs. According to Choirunnisa *et al*. [17], the 14-18% CP had 9% effect on carcass production on lambs. The absence of differences in meat-bone and meat-fat ratio was due to the absence of differences in growth rates that were indicated by ADG. Theoretically, excess intake of CP reported by Kearl [15] can increase ADG 70 g/day, but the intake only gives a difference of ADG 10-15 g/day. It indicates that there is excess protein diverted for fat synthesis. However, the excess did not give a difference to the fat. It was assumed that the capacity of cells to accommodate nutrients was close to the maximum level. It was confirmed by the finding of this research that ADG in this study was higher than the results obtained by Wildeus *et al*. [18] using Katahdin lambs (small frame) fed 17.5% CP which reported that the optimal ADG was 131 g/day.

### Nutrient intake and digestibility

Theoretically, the increase in CP level increases digestibility and intake of feed because the higher protein supply is capable of providing more substrate for the formation of rumen microbes that play a role in digesting feed [19]. However, in this study, the increase in CP level did not affect the digestibility of DM. It is because the increase in CP in this study was not followed by the increase in energy (iso energy), thus causing that the microbes which were formed were similar. It can be confirmed from the value of allantoin (Table-6). According to Pathak [20] and Jetana *et al*. [21], the optimizing of CP in forming rumen microbes needs to be balanced with energy content in the feed. The similar digestibility of the feed causes a similar flow rate of feed and results in a similar intake of the feed. Thus, the different CP levels cause the digestibility and intake of DM and nutrients contained to be similar except for the CP that was intentionally differentiated (Table-7). The result was agreed with the study of lambs fed 13-17% CP by Dabiri and Thonney [8], who found that intake of DM, OM, EE, NDF, and ADF was similar, while CP intake increased with increasing CP level. The increase in CP digestibility was similar to that of the study of Haddad *et al*. [2] who reported that increasing CP level from 10% to 18% CP linearly increases CP digestibility in lambs.

**Table-6:** Nitrous oxide emissions related to body weight gain and meat production.

Item	Treatment			SEM	p-value

	CP 14%	CP 16%	CP 18%		
N emission/kg ADG	110.5	124.9	123.9	0.40	0.740
N emission/kg carcass	5.0	4.6	3.6	0.98	0.364
N emission/kg meat	2.5	2.6	2.4	0.08	0.994
N emission/kg meat protein	65.7	57.5	46.7	13.26	0.396
N_2_O emission/kg ADG	2.2	2.5	2.5	0.40	0.742
N_2_O emission/kg carcass	0.027	0.029	0.026	0.00	0.764
N_2_O emission/kg meat	0.36	0.36	0.36	0.08	0.994
N_2_O emission/kg meat protein	0.35	0.36	0.33	0.78	0.946

SEM=Standard error of means, ADG=Average daily gain, N_2_O=Nitrous oxide

**Table-7:** Nutrient intake and apparent digestibility.

Item	Treatment			SEM	p-value

	CP 14%	CP 16%	CP 18%		
Intake					
DM, g	1274.5	1291.3	1265.1	1.16	0.974
DM intake, % BW	5.7	5.8	6.0	0.41	0.692
OM, g	911.1	928.5	911.7	83.52	0.972
CP, g	170.4^b^	201.1^ab^	223.7^a^	17.49	0.040
EE, g	50.6	48.8	45.0	4.45	0.474
NDF, g	696.4	681.5	648.6	62.09	0.740
ADF, g	439.7	430.3	412.5	39.24	0.786
NFE, g	499.6	491.9	464.2	44.66	0.716
Nutrients digestibility (%)					
DM	51.3	48.0	55.2	5.24	0.422
OM	50.8	49.1	55.5	5.33	0.493
CP	68.0^b^	69.6^b^	76.7^a^	3.14	0.048
EE	76.8	83.8	74.7	5.00	0.219
NDF	34.0	32.9	33.7	6.46	0.984
ADF	21.5	23.6	27.7	5.41	0.539
NFE	70.7	68.8	72.4	4.62	0.740

SEM=Standard error of means, DM=Dry matter, OM=Organic matter, CP=Crude protein, NDF=Neutral detergent fiber, ADF=Acid detergent fiber, NFE=Nitrogen free extract.

a,b Means with different superscripts in the same row differ significantly (p<0.05)

### Nitrogen balance and microbial efficiency

Nitrogen intake increased with increasing CP levels (p<0.05), but N excretion in urine and feces was similar (p>0.05). Theoretically, from the result, the N retention will be different among treatment, but in fact, from this study, there was no significant difference (p=0.052 close to significance level p<0.05). Its phenomenon was caused by the feed of 18% CP which has a higher proportion of soya bean meal compared to the feed 16% CP; theoretically, it would increase N excretion in urine. However, the results of the study showed that numerically N excretion in urine in 18% CP is lower than 16% CP. This is presumably due to the faster feed passage rate on 18% CP affected by the higher of feed intake in 18% CP compared to 16% CP (6.0% BW vs. 5.7% BW). The higher feed intake, the faster feed flow rate to leave the rumen; therefore, the process of degradation of feed in the rumen was shorter [22]. It causes N excretion in urine in 18% CP lower than compared to 16% CP. Fecal N excretion also has a similar pattern to urinary N excretion, in which fecal N excretion in CP 18% was lower compared to 16% CP. It was due to the effect of the fish meal (undegraded) whose the proportion was higher in 18% CP compared to 16% CP. The fish meal was easier to digest and absorb so that it reduces N excretion through feces. This reason was supported by the data of fecal N excretion in 18% CP which was lower (p<0.05) compared to CP 16% and 14%. The result of this study was similar to Dabiri and Thonney [8] who reported that CP level was not affected on N retention in their study on lambs fed 13-17% CP.

Increasing CP level increases N supply. It is theoretically able to increase microbial protein synthesis. However, in this study, an increase in N supply was not able to increase microbial protein synthesis presumably because N supply was not balanced with energy supply, causing the similar microbes to form. This study used iso energy with 60% of total digestible nutrients (TDN); therefore, it will differ on CP/TDN ratio (0.23 vs. 0.26 vs. 0.30 for 14, 16, and 18% CP level, respectively). This result confirmed to the result [23] who reported that CP/TDN ratio ranging from 0.23 to 0.29 resulted in a similar number of microbial protein supply. The optimizing of feed proteins in forming rumen microbes needs to be balanced with energy [17,21].

### ADG, carcass production, and meat productions related to nitrous oxide emissions

The effect of CP level on N_2_O was similar among the treatments (p>0.05). The similarity of N_2_O emissions can be explained by similarity on the total N excreted through feces and urine. Menezes *et al*. [24] stated that the N_2_O formed was determined by the N excretion in the feces and urine. The results of this study were similar to a study reported by Menezes *et al*. [24] on Nelore bull cattle fed 10-14% protein levels that N_2_O emissions were similar.

In this study, the effect of CP level was not significant on the N_2_O emissions related to ADG, carcass production, meat production, and meat protein produced. It is because CP level was not effected on N_2_O emissions, ADG, carcass production, meat productions as well as on meat protein produced. Therefore, N_2_O emissions related to ADG, carcass production, meat production, and meat protein produced were similar. The result of this study was similar to Menezes *et al*. [24] who reported that the 10-14% of CP did not effect on the emissions related to ADG and carcass production.

## Conclusion

The increase of CP level up to 18% did not affect productivity, N emissions, and efficiency of emissions per unit product because the increase of CP was not balanced by energy content in feed. Therefore, for further research, the productivity and emissions need to be clarified by increasing the level of protein that was balanced with energy content in the feed.

## Authors’ Contributions

AP carried out the study and wrote the manuscript. EP, ER, and APu participated in the drafting manuscript. All authors planned and conducted the study. All authors corrected the manuscript read and approved the final manuscript.

## References

[1] Ma T, Wang B, Zhang N, Tu Y, Si B, Cui K, Diao Q (2017). Effect of protein restriction followed by realimentation on growth, nutrient digestibility, ruminal parameters, and transporter gene expression in lambs. Anim. Feed Sci. Technol.

[2] Haddad S.G, Nasr R.E, Muwalla M.M (2001). Optimum dietary crude protein level for finishing Awassi lambs. Small Rumin. Res.

[3] Santos R.S, Ribeiro K.G, Filho S.C.V, Pereira O.G, Villela S.D.J, Rennó L.N, Silva J.L (2015). Effects of diets with high and low protein contents and two concentrate levels in santa ines âtexel lambs. Lives Sci.

[4] Estrada-Angulo A, Castro-Pérez B.I, Urías-Estrada J.D, Ríos-Rincón F.G (2018). Influence of protein level on growth performance, dietary energetics and carcass characteristics of pelibuey Katahdin lambs finished with isocaloric diets. Small Rumin. Res.

[5] Kiran D, Mutsvangwa T (2009). Nitrogen utilization in growing lambs fed oscillating dietary protein concentrations. Anim. Feed Sci. Technol.

[6] Beauchemin K.A, McClelland L.A, Jones S.D.M, Kozub G.C (1995). Effects of crude protein content, protein degradability and energy concentration of the diet on growth and carcass characteristics of market lambs fed high concentrate diet. Can. J. Anim. Sci.

[7] Ludden P.A, Wechter T.L, Hess B.W (2002). Effects of oscillating dietary protein on nutrient digestibility, nitrogen metabolism, and gastrointestinal organ mass in sheep. J. Anim. Sci.

[8] Dabiri N, Thonney M.L (2004). Source and level of supplemental protein for growing lambs. J. Anim. Sci.

[9] Kebede G (2015). Optimum dietary crude protein level for fattening yearling arsi-bale lambs. World J. Agric. Sci.

[10] AOAC (2005). Official Methods of Analysis.

[11] Van Soest P.J, Robertson J.B, Lewis B.A (1991). Methods for dietary fiber, neutral detergent fiber and non-starch polysaccharides in relation to animal nutrition. J. Dairy Sci.

[12] Chen X.B, Gomes M.J (1992). Estimation of Microbial Protein Supply to Sheep and Cattle based on Urinary Excretion of Purine Derivatives An Overview of Technical Details (Occasional Publication).

[13] IPCC (2006). Emissions from livestock and manure management. Guidelines for National Greenhouse Inventories.

[14] Gomez K.A, Gomez A.A (1984). Statistical Procedures for Agricultural Research.

[15] Kearl L.C (1982). Nutrient Requirements of Ruminants in Developing Countries.

[16] Chay-Canul A.J, Espinoza-Hernandez J.C, Aguilar-Perez C.F, Ayala-Burgos A.J, Maga J.G, Chizzotti M.L, Ku-Vera J.C (2014). Relationship of empty body weight with shrunken body weight and carcass weights in adult Pelibuey ewes at different physiological states. Small Rumin. Res.

[17] Choirunnisa R, Prima A, Luthfi N, Arifin M, Sutaryo, Purnomoadi A (2016). Correlation between Crude Protein Levels in the Diets and Carcass Weight and Carcass Percentage in Thin Tailed Lambs.

[18] Wildeus S, Turner K.E, Collins J.R (2007). Growth, intake, diet digestibility, and nitrogen use in three hair sheep breeds fed alfalfa hay. Small Rumin. Res.

[19] Chun-Tao Y, Bing-Wen S.I, Qi-Yu D, Hai J.I.N, Shu-Qin Z, Yan T.U (2016). Rumen fermentation and bacterial communities in weaned Chahaer lambs on diets with different protein levels. J. Integr. Agric.

[20] Pathak A.K (2008). Various factors affecting microbial protein synthesis in the rumen-a review. Vet. World.

[21] Jetana T, Abdullah N, Halim R.A (2000). Effects of energy and protein supplementation on microbial-N synthesis and allantoin excretion in sheep fed guinea grass. Anim. Feed Sci. Technol.

[22] Lee S.K, Youngil K, Youngkyoon O, Wansup K (2010). Effects of feeding methods of total mixed ration on behavior patterns of growing Hanwoo steers. Asian Aus. J. Anim. Sci.

[23] Chanthakhoun V, Wanapat M, Berg J (2012). Level of crude protein in concentrate supplements influenced rumen characteristics, microbial protein synthesis and digestibility in swamp buffaloes (*Bubalus bubalis*). Lives Sci.

[24] Menezes A.C.B, Filho S.C.V, Silva L.F.C, Pacheco M.V.C, Pereira J.M.V, Rotta P.P, Rennó L.N (2016). Does a reduction in dietary crude protein content affect performance, nutrient requirements, nitrogen losses, and methane emissions in finishing Nellore bulls?. Agric. Ecosyst. Environ.

